# Kidney Dysfunction, Hepatic Impairment, and Lipid Metabolism Abnormalities in Patients with Precapillary Pulmonary Hypertension

**DOI:** 10.3390/diagnostics14161824

**Published:** 2024-08-21

**Authors:** Dragos Gabriel Iancu, Andreea Varga, Liviu Cristescu, Robert Adrian Dumbrava, Florin Stoica, Diana Andreea Moldovan, Radu Adrian Suteu, Ioan Tilea

**Affiliations:** 1 Doctoral School, G.E. Palade University of Medicine, Pharmacy, Science and Technology of Targu Mures, 540142 Targu Mures, Romania; dragos-gabriel.iancu@umfst.ro (D.G.I.); liviu.cristescu@umfst.ro (L.C.); robert-adrian.dumbrava@umfst.ro (R.A.D.); stoica.florin.23@stud.umfst.ro (F.S.); diana.moldovan@umfst.ro (D.A.M.); 2 Department of Internal Medicine II, Emergency Clinical County Hospital, 540042 Targu Mures, Romania; 3 Faculty of Medicine, G.E. Palade University of Medicine, Pharmacy, Science and Technology of Targu Mures, 540142 Targu Mures, Romania; ioan.tilea@umfst.ro; 4 Department of Cardiology II, Emergency Clinical County Hospital, 540042 Targu Mures, Romania; 5 Department of Cardiology I, The Emergency Institute for Cardiovascular Diseases and Transplantation, 540136 Targu Mures, Romania; radu.suteu@umfst.ro

**Keywords:** pulmonary arterial hypertension, chronic thromboembolic pulmonary hypertension, kidney dysfunction, hepatic impairment, lipid metabolism abnormalities

## Abstract

Background: Pulmonary hypertension (PH) is a global health issue that has profound medical and research implications. Methods: This retrospective study examined changes in renal and liver function, as well as lipid metabolism, over a 12-month period in 49 adult patients with pulmonary arterial hypertension (PAH) and chronic thromboembolic pulmonary hypertension (CTEPH). All cases were admitted, managed, and followed up with in the PH Center, County Emergency Clinical Hospital of Targu Mures, Romania. Results: Kidney dysfunction was observed in 12.24% of cases at baseline, decreasing to 8.16% at 12 months, and CTEPH patients were more affected. In particular, CTEPH patients exhibited an improvement in renal function, confirmed by an increase in their glomerular filtration rates. Hepatic impairment was present in 57.14% of subjects at baseline, declining to 42.86% at 12 months, with significant improvements noted in the PAH group. Lipid metabolic dysregulations were experienced by 22.45% of all patients at baseline, decreasing to 16.33% at 6 months, with a slow elevation to 24.49% at 12 months, but with no statistically significant differences. Pharmacological regimens were adjusted in accordance with the PH groups, a patient’s functional and clinical response, and laboratory tests. Conclusions: Our results demonstrate the multi-organ damage in PH and the importance of individualized treatment approaches.

## 1. Introduction

Pulmonary hypertension (PH) is defined as a cardiopulmonary disease, in which elevated blood pressure in the pulmonary circulation affects the function of the right ventricle (RV) and which can later contribute to RV failure [1]. A definitive PH diagnosis is confirmed by the reports of right cardiac catheterization as an increase in the mean pulmonary arterial pressure (mPAP) ≥ 20 mmHg at rest [1]. The currently available data show that the normal mPAP at rest is 14 ± 3 mmHg, with an upper limit of 20 mmHg. Pulmonary hypertension can be classified into two subcategories based on the pulmonary artery wedge pressure (PAWP) and pulmonary vascular resistance (PVR). Thus, a PAWP value ≤15 mmHg and a PVR > 2 WU defines precapillary PH, and a PAWP > 15 mmHg alongside a PVR ≤ 2 WU indicates isolated post-capillary PH [1]. In an intriguing development, in 2019, Simonneau et al. refined the 2015 European Society of Cardiology/European Respiratory Society (ESC/ERS) definition of pulmonary hypertension. At that time, they proposed a new threshold of PAPm > 20 mmHg and the need for pulmonary vascular resistance (PVR) ≥ 3 WU to indicate the presence of precapillary pulmonary hypertension [2]. Current ESC/ERS guidelines demarcate pulmonary hypertension into five major groups: pulmonary arterial hypertension; pulmonary hypertension associated with left heart disease; pulmonary hypertension associated with lung disease; pulmonary hypertension associated with pulmonary artery obstructions; and pulmonary hypertension with unclear and/or multifactorial mechanisms [1,3,4].

Increasing evidence from recent studies has shown that PH is not merely a cardio-pulmonary disease but a multi-organ disease, represented by malfunctions of the circulatory system, central and peripheral nervous system, kidney and liver function, and lipid metabolism; skeletal myopathy; and effects on the immune response [5,6].

Currently, the precise relationship between PH, right ventricular failure, and the deterioration of renal function has not been established [7]. What has been proven is that many factors lead to the development and worsening of pulmonary hypertension, and the appearance of renal dysfunction will lead to a volume overload that will also cause increased pulmonary arterial pressure. The renal function declining is linked to hemodynamic changes caused by the volume overload associated with PH. If the kidney damage persists or the underlying disease worsens, these changes in renal function may become permanent [8,9,10]. It has been observed that in patients with pulmonary hypertension and chronic kidney disease, an increased value of right atrium pressure (RAP), a low cardiac index (CI), or an increased value of mPAP is correlated with a poor prognosis [9,11].

Many PH-targeted therapies possess attributes that protect the renal function due to their vasoactive properties. A ubiquitous characteristic of kidney failure in all manifestations of PH is cardiorenal syndrome (CRS), a clinical condition wherein the management of heart or kidney failure is constrained by the exacerbation of heart or kidney function [12].

The cornerstone of contemporary PH-targeted therapy primarily involves potent vasoactive agents such as phosphodiesterase type 5 inhibitors (PDE5is), endothelin receptor antagonists (ERAs), and prostacyclin analogues, all of which can impact the highly vascularized kidneys. Sildenafil, the first PDE5i studied in PH, demonstrates a range of nephroprotective effects [13]. A small randomized controlled trial suggested that bosentan and macitentan possess renal-protective effects that are independent of their blood-pressure-lowering properties [14]. Riociguat represents a novel therapeutic approach for PH by stimulating soluble guanylate cyclase in vascular smooth muscle cells (SMCs). As of now, there is a lack of clinical data regarding the impact of soluble guanylate cyclase stimulators on kidney disease. Although riociguat is cleared renally, it has not undergone evaluation in patients with end-stage renal disease [15,16].

The exact prevalence of kidney dysfunction in PH varies depending on the subtype, but it is generally recognized to be high, ranging from 4% to 36% [17,18,19]. An individual’s serum creatinine measurement varies and somewhat reflects changes in the glomerular filtration rate, roughly indicating kidney function and injury [20]. The bidirectional relationship between pulmonary hypertension and chronic kidney disease is well documented in previous studies [21,22].

In PAH patients, a baseline estimated glomerular filtration rate (eGFR) of less than 60 mL/min/1.73 m^2^ and a subsequent decline of 10% in eGFR are substantial predictors of poorer clinical outcomes [23].

Because of its anatomical and physiological relation with the right ventricle (RV), the liver is among the first organs to be affected by the onset of right ventricular failure (a mechanism caused by volume overload of the RV that leads to chronic congestive liver disease). There is a close relationship between the liver, lungs, and heart, so a liver function injury can have various effects on the pulmonary vascular system [5].

The liver’s susceptibility to vascular insult is attributable to its dual blood supply and its elevated metabolic demands. Circulatory disturbances primarily affecting the liver include congestion resulting from right heart failure, ischemic injury due to low-cardiac-output states, or a combination of both factors [24,25]. Patients experiencing acutely decompensated heart failure and reduced cardiac output frequently exhibit a mixed biochemical liver profile, characterized by signs of hepatocellular injury such as elevated AST and ALT levels, alongside increases in cholestatic liver enzymes, including bilirubin and alkaline phosphatase (ALP) [26].

Mild elevations in cholestatic biochemical markers, including bilirubin, gamma-glutamyl transferase (GGT), AST, ALT, and ALP, are detected in 17 to 77% of patients with stable congestive heart failure (CHF) [27,28,29].

Compared with LHD, there is a paucity of research regarding liver test abnormalities in PH. Similarly, in the absence of acute decompensated heart failure, the predominant biochemical pattern of liver injury in PH patients is characterized by cholestasis [30,31,32].

Moreover, certain commonly utilized agents belonging to a class of chronic pharmacologic pulmonary vasodilators, such as endothelin receptor antagonists, may induce elevations in AST and ALT levels, occasionally warranting discontinuation [33].

Despite its significant impact on mortality and its close association with right-sided hemodynamics, hepatic impairment in PH patients is likely underreported in clinical trials and large registries. This particular aspect requires further detailed investigation to comprehensively understand pulmonary hypertension’s relationship with liver dysfunction.

Currently, five pharmacological classes are approved for the treatment of PH: prostanoids, prostacyclin receptor agonists, PDE5is, soluble guanylate cyclase stimulators (SGCSs), and endothelin receptor antagonists (ERAs). Except for epoprostenol, most PH-targeted therapies undergo at least partial hepatic metabolism [34]. Significant hepatotoxicity from PH-targeted therapies is uncommon. When hepatotoxicity does occur, it typically presents as a hepatocellular injury pattern characterized by elevated transaminase levels. Of the three prostanoids used to manage PH, iloprost and treprostinil are metabolized by the liver, and their clearance is reduced in patients with hepatic impairment [35]. PDE5is undergo hepatic metabolism. However, neither sildenafil nor tadalafil have been thoroughly studied in individuals with severe liver disease (Child–Pugh class C), and their use is generally advised against in this group [36].

ERAs are metabolized by the liver, but bosentan is unique, as it acts as both a substrate and an inducer of the cytochrome system [37]. A recent meta-analysis indicated that bosentan carries the highest risk of hepatotoxicity compared with ambrisentan and macitentan [38].

Riociguat, an SGCS, undergoes hepatic metabolism, and liver impairment can result in increased drug concentrations. Therefore, riociguat is not advised for patients with severe hepatic impairment (Child–Pugh class C). It is also important to closely monitor patients for possible drug–drug interactions while they are on riociguat [39].

Patients with PH are known to have abnormal circulating lipid and lipoprotein concentrations. The underlying reasons for these abnormalities and their clinical significance remain unclear. Available reports indicate that individuals with PH often exhibit reduced levels of low-density lipoprotein (LDL), high-density lipoprotein (HDL), and chylomicrons in their circulation [40,41]. Several studies have addressed lipid homeostasis disorder in PAH patients in the past decade. Recent studies suggest an aberrant disorder of lipid homeostasis at the cellular but also systemic level [6,42,43]. Metabolic syndrome has been identified in a large proportion of patients with pulmonary hypertension, and it has features that are characteristic of patients with insulin resistance and abnormalities in their lipid metabolism [44,45,46]. Also, a reduction in the LDL cholesterol levels is considered a prognostic biomarker in PAH patients [41]. In CTEPH patients, low levels of HDL cholesterol are related to the dysfunction of the right ventricle, but they do not correlate with the disease severity and prognosis as suggested by Khirfan et al. [47].

The differential distribution of circulating lipids in PH may stem from various factors. Inflammation, for instance, is recognized as a key modulator of the lipid metabolism, implicated not only in PH but also in a spectrum of inflammatory conditions such as rheumatoid arthritis, psoriasis, lupus, and inflammatory bowel disease [48]. Moreover, inflammation constitutes a pivotal component in the genesis and progression of PH, often occurring concomitantly with various inflammatory disorders. Extensive research has demonstrated elevated levels of circulating cytokines, chemokines, tissue factors, and C-reactive proteins in PH patients, shedding light on the intricate interplay between inflammation and lipid dysregulation in this context [49].

This retrospective study aimed to identify the primary changes in renal, liver, and lipid metabolism function in two distinct groups of pulmonary hypertension patients—PAH and CTEPH—and to identify possible specific predictive parameters of these alterations, thereby enabling the initiation of appropriate treatment to improve their prognosis.

## 2. Materials and Methods

The present study is a retrospective analysis conducted at a single reference center for pulmonary hypertension. This research encompasses a cohort of 49 PAH and CTEPH patients who were followed up with from September 2015 to January 2024 at the Clinical County Hospital in Targu Mures, Romania. This institution has been part of the National Program for Pulmonary Hypertension Treatment since 2015. The inclusion criteria for this study comprised a confirmed diagnosis and clinical classification of PAH and CTEPH cases in accordance with the ESC/ERS 2015 and 2022 guidelines existing at that time. This research was approved by the Ethics Committee of the G.E. Palade University of Medicine, Pharmacy, Science and Technology of Targu Mures, Romania (approval number 1283/25.02.2021).

This study categorized patients into two major groups: those with pulmonary arterial hypertension and those with chronic thromboembolic pulmonary hypertension. The PAH group comprised 35 individuals, further classified by etiology: 7 patients had idiopathic PAH (IPAH), 4 patients had PAH associated with connective tissue disease, 1 patient had PAH related to portal hypertension (PoPH), and 23 patients had PAH secondary to congenital heart disease (CHD-associated PAH). The CTEPH group consisted of 14 patients.

The inclusion criteria were being over 18 years of age and biochemical testing of serum creatinine, eGFR (calculated using CKD-EPI Equation for Glomerular Filtration Rate), total bilirubin, GGT, serum aminotransferases (AST, ALT), ALP, cholesterol, and triglycerides.

Patients were allowed to continue other background therapy with other drugs. They were monitored for a period of twelve months, and regular assessments were scheduled at baseline and six and twelve months.

Blood samples were provided by all participants after 12 h of fasting. Biochemical blood tests were performed using a Konelab Prime 60i (Thermo Fisher Scientific Inc., Waltham, MA, USA) analyzer in the local ISO-15189-certified laboratory. The reference value ranges were as follows: serum creatinine at 0.7–1.2 mg/dL; eGFR > 60 mL/min/1.73 m^2^; total bilirubin at 0.3–1.2 mg/dL; gamma-glutamyl transferase at 11–50 U/L; AST at 5–45 U/L; ALT at 5–45 U/L; ALP at 100–300 U/L; total cholesterol at 2.8–5.2 mmol/L; and triglycerides at 0.55–1.9 mmol/L.

Kidney dysfunction was assigned by an eGFR value less than 60 mL/min/1.73 m^2^. Hepatic impairment was characterized by elevated levels of hepatic transaminases (defined as a two-fold increase above the upper limit of the normal range), bilirubin, or alkaline phosphatase. Furthermore, lipid metabolism dysfunction was defined by elevated levels of triglycerides or cholesterol above the laboratory’s normal reference range.

Demographic characteristics, functional classes according to the World Health Organization (WHO), 6 min walk test (6MWT) results, electrocardiographic patterns and echocardiographic parameters, NT-proBNP levels, and pharmacological regimens were collected at the time of inclusion in the National Program for Pulmonary Hypertension Treatment.

At the baseline and throughout the abovementioned time frames, all patients were tested for viral hepatitis markers to exclude a potential cause of liver dysfunction. Additionally, the included patients did not receive hepatotoxic or nephrotoxic medication, and abdominal ultrasound was performed to exclude potential liver or kidney damage (e.g., polycystic kidney disease).

The study period commenced at the moment of a patient’s inclusion in the Pulmonary Hypertension Program Treatment and extended until January 2024. Each patient was evaluated every three months, or more frequently if their clinical condition worsened, in accordance with the ESC/ERS 2015 and 2022 guidelines. Throughout the follow-up period, assessment included an evaluation of the WHO functional class, 6MWT, resting ECG, and biochemical testing. A cardiac ultrasound was typically performed biannually or upon the deterioration of symptoms and was conducted by two experienced independent operators utilizing a GE Vivid™ E9 ultrasound system (GE Vingmed Ultrasound AS, Horten, Norway).

Statistical analyses were completed using R Studio statistical software (R Core Team (2020). R: A language and environment for statistical computing. R Foundation for Statistical Computing, Vienna, Austria. URL: https://www.R-project.org/ (accessed on 10 April 2024)). Parametric continuous data are presented as the mean ± standard deviations, while non-parametric continuous variables are reported as the median (interquartile range). Binary variables are expressed as counts (percentages). The Shapiro–Wilk test was used to assess normality. Statistical differences between variables were evaluated using unpaired and paired *t*-tests, chi-square tests (χ^2^-value), and the Mann–Whitney test, as appropriate. Correlations were determined using Spearman’s rank correlation coefficient for non-parametric variables. A *p*-value of ≤0.05 was considered statistically significant.

## 3. Results

This study included 49 patients aged 49.41 ± 18.68 years (61.22% women). Pulmonary arterial hypertension was diagnosed in 71.42% of cases (see Table 1).

In our study, the median WHO functional class for patients with PAH improved from 3.00 (IQR: 2.00–3.00) at baseline to 2.00 (IQR: 2.00–3.00) after 12 months. Similarly, CTEPH patients showed an improvement from 2.50 (IQR: 2.00–3.00) to 2.00 (IQR: 2.00–3.00) during the same period, with no significant differences between the groups. Moreover, the severity of right heart failure exhibited a marked improvement, with the percentage of PAH patients classified as regular increasing from 54.28% to 71.42%, while in CTEPH patients, this proportion rose from 42.85% to 64.28% over a 12-month period. The interval from the initial diagnosis of pulmonary hypertension to study enrollment did not demonstrate a statistically significant difference between the two groups (see Table 2).

The prevalence of non-PH related factors showed no significant differences between PAH and CTEPH patients, except for deep vein thrombosis (*p* < 0.0001). Conditions such as type 2 diabetes mellitus (T2DM), hypertension (HTN), CHF, atrial fibrillation, thyroid disease, obstructive sleep apnea, lung disease, chronic obstructive pulmonary disease (COPD), asthma, and SARS-CoV-2 infection had similar prevalence rates between the two groups (see Table 3).

**Figure 1:**
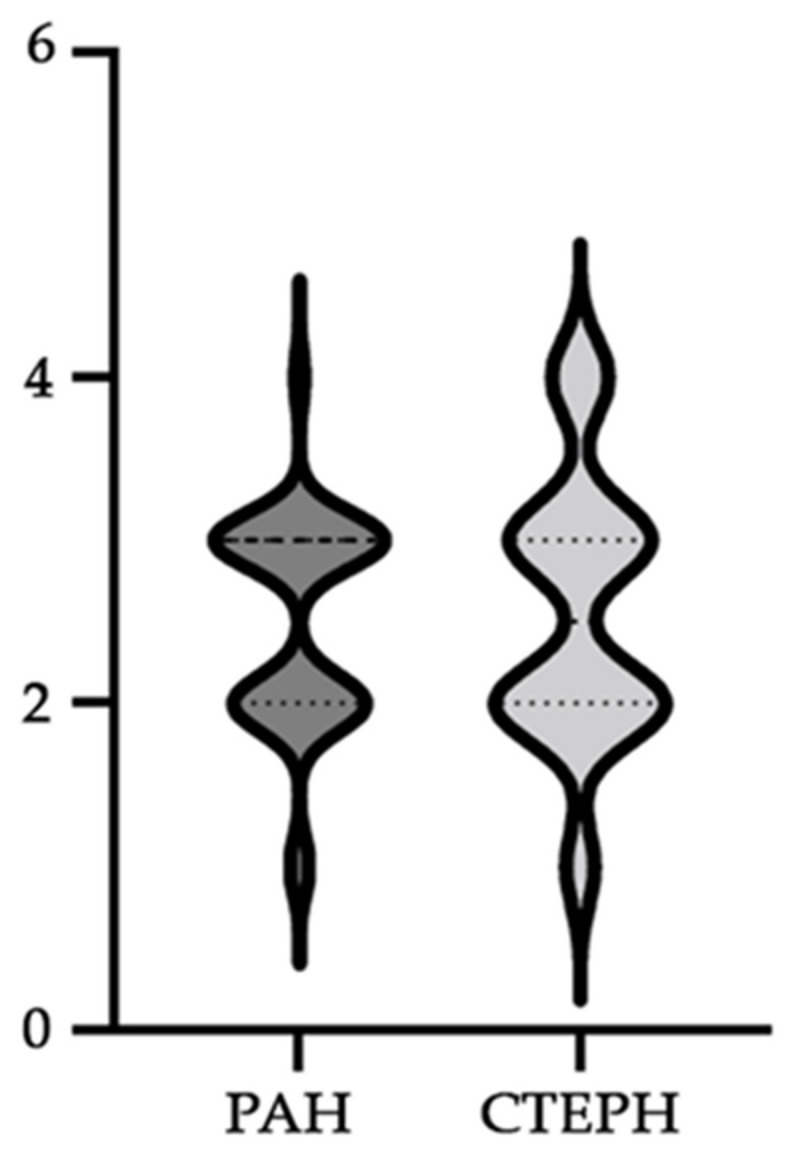
A frequency bar graph depicting the World Health Organization (WHO) functional class values within the PAH and CTEPH groups is illustrated.

Evaluating the WHO functional class, no significant statistical correlations were detected between the two studied groups. Within the PAH group, 51.43% belonged to WHO functional class III, while 40% belonged to class II. In contrast, within the CTEPH group, the proportion of membership in WHO functional class III was 35.71%, with 42.86% belonging to class II (see Figure 1).

At baseline, renal dysfunction was present in 12.24% (*n* = 6) of cases, hepatic impairment in 57.14% (*n* = 28), and lipid homeostasis disorder in 22.45% (*n* = 11). At six months, renal dysfunction was present in 16.33% (*n* = 8) of cases, liver dysfunction in 53.06% (*n* = 26), and lipid metabolism disorders in 12.24% (*n* = 6). At the twelve-month follow-up, renal dysfunction was present in 8.16% (*n* = 4) of cases, liver dysfunction in 42.86% (*n* = 21), and lipid metabolism abnormalities in 24.49% (*n* = 12). The statistical analyses of the obtained data are depicted in Table 4.

A statistically significant difference was identified in the frequencies of renal dysfunction occurrence between the PAH and CTEPH groups (2.86–8.57% within the PAH group, compared with 35.71% within the CTEPH group). Additionally, an improvement in the glomerular filtration rate was noted in the CTEPH patient group, although this improvement did not reach statistical significance.

Analyzing the percentage of patients with kidney dysfunction included in our study, we found that at the time of inclusion, it was 16.66% (*n* = 6); at 6 months, it was 16.32% (*n* = 8); and at 12 months, it was 8.16% (*n* = 4). Furthermore, renal dysfunction was statistically significantly more prevalent (*p* < 0.05) among patients with CTEPH compared with those with PAH at all three time points of this study.

Diuretic therapy is commonly prescribed as standard care for patients with PH. In our study, 87.76% (*n* = 43, PAH = 29, CTEPH = 14) of patients were receiving diuretic therapy at the time of enrollment, 95.92% (*n* = 47, PAH = 33, CTEPH = 14) at 6 months, and 95.92% (*n* = 47, PAH = 33, CTEPH = 14) at 12 months.

In the context of hepatic impairment, in the PAH group, a statistically significant progressive improvement was observed throughout the study duration (*p* = 0.0027). Among patients included in this study at baseline, 16 out of 28 had liver dysfunction due to cholestasis syndrome, most of them from group 4.1 ESC/ERS clinical classification (20.69%, *n* = 6) and group 1.4.4 ESC/ERS clinical classification (24.13%, *n* = 7). At 6 months, 14 patients presented liver dysfunction due to cholestasis syndrome (2 cases associated with hepatocytolysis syndrome), and 12 months after inclusion, 10 patients presented dysfunction due to cholestasis syndrome (1 case associated with hepatocytolysis syndrome).

At the time of inclusion in this study, 57.14% of patients were at the upper limit of normal in their bilirubin, GGT, AST, ALT, and ALP values. Furthermore, at six and twelve months, 53.06% and 42.86%, respectively, of the cases still exhibited such elevations.

The total bilirubin level of patients at the initial moment exceeded the upper limit of normal in 32.65% of cases (*n* = 16, PAH = 10, CTEPH = 6), at six months in 28.57% (*n* = 14, PAH = 10, CTEPH = 4), and at twelve months in 20.41% (*n* = 10, PAH = 4, CTEPH = 6). Additionally, the total bilirubin level did not show a statistically significant difference between the groups of patients with PAH and CTEPH at the time of inclusion or at 6 months. However, at 12 months, it was statistically significantly lower (*p* = 0.0384).

The AST and ALT levels of patients at the time of study inclusion exceeded the normal values in 2% of cases (*n* = 1, CTEPH), at six months in 8.16% (*n* = 4, PAH = 2, CTEPH = 2), and at twelve months in 6.12% (*n* = 3, PAH). The AST and ALT levels did not show statistically significant differences between the groups of patients with PAH and CTEPH at all moments of follow-up (*p* > 0.05).

In our study, 4.08% (*n* = 2) of patients on bosentan experienced elevated aminotransferase levels.

Regarding ALP, 12.24% (*n* = 6) of patients receiving ERA treatment presented elevated values of this biomarker.

At six and twelve months, irrespective of the specific medication regimens, a statistically significant higher level of ALP was observed in CTEPH patients compared with those with PAH (*p* < 0.05). Additionally, the GGT value was statistically significantly higher in the group of CTEPH patients at all three monitored time points (*p* < 0.05).

At the time of inclusion in this study, nine patients (18.36%) were hypercholesterolemic (three of them with associated hypertriglyceridemia), with a decrease in the number of cases (3–6.12%) at 6 months (hypertriglyceridemia was associated with two cases), and at 12 months, eight (16.32%) patients presented with hypercholesterolemia (two combined with hypertriglyceridemia). No statistically significant differences were observed regarding lipid metabolism determinants. An explanatory factor may lie in the concurrent administration of lipid-lowering agents, mainly an atorvastatin dose, adjusted in accordance with the total cardiovascular risk of patients.

**Figure 2:**
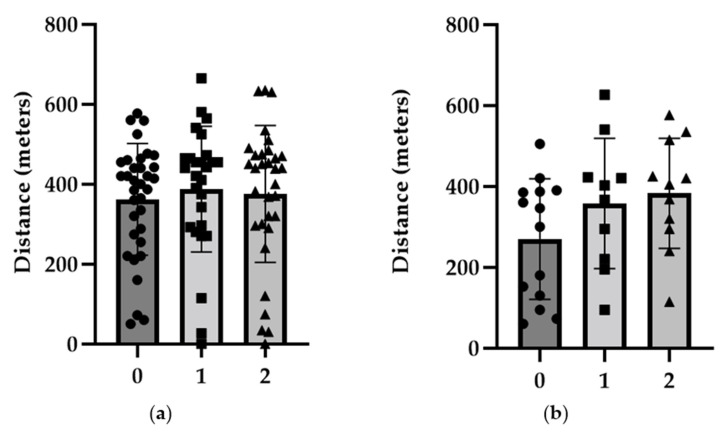
Values of the 6MWT comparing baseline (0), follow-up at 6 months (1), and follow-up at 12 months (2) within the PAH (**a**) and CTEPH (**b**) groups, with the exposure of individual values for each moment.

During the 12-month study period, an improvement in the 6MWT performance within the CTEPH group of 22.27% from baseline to the 12-month evaluation was noticed. In comparison, the observed difference in the PAH group was only 8.88% (see Figure 2).

**Figure 3:**
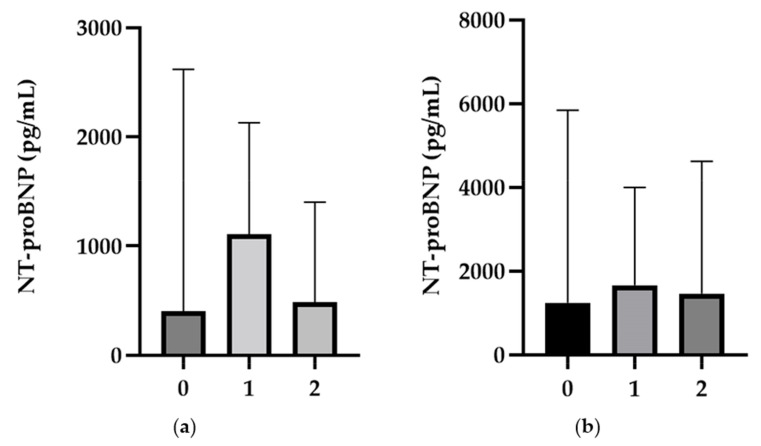
A comparative illustration of the median NT-proBNP values accompanied by the interquartile range (IQR) for the three studied time points within the PAH (**a**) and CTEPH (**b**) groups. 0, baseline; 1, follow-up at 6 months; 2, follow-up at 12 months.

Additionally, a significantly lower NT-proBNP value was observed in the PAH group compared with the CTEPH one, which maintained elevated NT-proBNP levels at baseline, 6 months, and 12 months, respectively. There was a statistically significant difference in NT-proBNP levels between PAH and CTEPH patients at the 12-month follow-up (*p* = 0.045) (see Figure 3).

Regarding the four-strata risk assessment, the analysis reveals that there are no statistically significant differences in the mean risk scores of PAH and CTEPH across the various time points evaluated. Both inter-group (PAH vs. CTEPH) and intra-group (within PAH and CTEPH across different time points) comparisons yielded non-significant *p*-values, suggesting that the observed differences in mean risk scores over time are not statistically significant.

The medication administered to patients over the duration of this study is comprehensively detailed below in Table 5.

During the twelve months of follow-up, specific medications for pulmonary hypertension (PDE5is, ERAs, SGCSs, or a combination of PDE5is and ERAs) did not lead to the onset of kidney, liver, or lipid metabolism dysfunction (*p* ≥ 0.05).

Following the initiation of targeted therapy for PH, a comparison of the frequencies of hepatic impairment at admission and at the 12-month follow-up demonstrated statistically significant differences (*p* < 0.001) in the PAH patient cohort. However, no significant differences were found concerning renal dysfunction in the same group. In contrast, for the CTEPH group, no statistically significant differences were identified in the frequencies of hepatic impairment and renal dysfunction at admission versus 12-month follow-up. Regarding the frequency of renal dysfunction in the CTEPH group, a *p*-value of 0.067 was obtained, suggesting that in a multicentric study with a larger sample size, this difference might reach statistical significance. No statistically significant correlations were observed between improvements in the 6-MWD and the aforementioned parameters.

Upon testing the patient groups, a statistically significant difference was noted in the SGCS regimen, with CTEPH patients receiving this drug significantly more frequently than those with PAH. Additionally, at this study’s baseline, individuals with CTEPH were statistically significantly more likely to be prescribed beta-blocker therapy compared with PAH patients (*p* = 0.0233). However, this disparity diminished over time due to medication adjustments tailored to each patient’s clinical requirements. The inclusion of other classes of medications and monitoring their effect throughout the study period did not show a statistical significance between the studied cohorts.

## 4. Discussion

This study offers comprehensive insights into kidney function, hepatic impairment, and abnormalities in lipid metabolism among specific groups of pulmonary hypertension patients, focusing on PAH and CTEPH. The findings highlight the dynamic nature of these parameters over a 12-month period, shedding light on potential implications for patient management and treatment strategies. In our cohort of precapillary pulmonary hypertension patients, pulmonary arterial hypertension was the predominant group, representing 71.42% of cases, followed by chronic thromboembolic pulmonary hypertension, which accounted for 28.58% of cases.

Advancements in the comprehension of PH and the emergence of novel therapies over recent decades have contributed to enhanced survival outcomes in patients diagnosed with PAH and CTEPH. However, despite these strides, PH continues to pose a significant clinical challenge with a discouraging prognosis. As the disease advances, it commonly precipitates dysfunction in various organs and systems, prominently affecting renal, liver, and lipid function, which has been correlated with heightened mortality rates. It is well known that pulmonary hypertension has been studied for several decades and that a variety of etiological factors determine the subgroups of this complex pathology, which makes this a special category, in which the continuous evaluation of the pathogenesis and progression of the disease is a challenge from a clinical point of view. In recent years, numerous important studies highlighting a significant link between biochemical markers and the pulmonary hypertension phenotype have been published [50]. Some of the biochemical markers frequently used in these studies, such as the number of erythrocytes, aminotransferases, creatinine, and uric acid, are generally increased in all subgroups of pulmonary hypertension, although these were analyzed predominantly in patients with IPAH. Pulmonary hypertension and chronic kidney disease are pathologies with poor results in the medium and long term [51,52].

The exact prevalence of kidney dysfunction in PH varies depending on the subtype, but it is generally recognized to be high, ranging from 4% to 36%. This rate is significantly higher than that in the general population, where kidney dysfunction is reported in about 0.6% of men and 0.3% of women. Factors such as older age, high systemic blood pressure, diabetes, and increased right atrial pressure are linked to kidney malfunction in PAH patients [17,18,19]. In our study, the results related to the prevalence of kidney dysfunction are consistent with those reported in the study conducted by Nickel et al. [17].

In patients with PAH, the baseline estimated glomerular filtration rate and a 10% decline in eGFR over time are significant predictors of clinical outcomes. A lower eGFR at baseline, specifically less than 60 mL/min/1.73 m^2^, is associated with worse survival rates compared with those with higher eGFR levels. Furthermore, a 10% decrease in eGFR from baseline is linked to increased mortality and a higher risk of hospitalization, underscoring the importance of monitoring renal function as part of managing PAH patients [23].

The REVEAL registry assessed 2716 patients with PAH, identifying that 4% of subjects exhibited renal dysfunction, albeit without a precise definition. In our study, conducted over a 12-month period from enrollment, an incidence of renal dysfunction between 8.16% and 16.66 was identified, indicating a discrepancy between our study’s findings and registry data [30].

From an earlier assessment using data from the REVEAL registry, renal failure was identified as a predictive factor that is associated with higher mortality rates. It was subsequently incorporated into the REVEAL Risk Score Calculator, although its definition was broad and left to the investigator’s interpretation. Within the Pulmonary Hypertension Connection registry, comprising 500 patients, those with serum creatinine (SCr) levels ranging from 1.0 to 1.4 mg/dL and levels exceeding 1.4 mg/dL demonstrated higher mortality risks of 1.65 and 2.54, respectively, in comparison to individuals with baseline SCr levels below 1.0 mg/dL. Moreover, within the same cohort, elevated SCr levels correlated with an elevated mean right atrial pressure and reduced cardiac index, suggesting a connection between elevated SCr levels and a poorer hemodynamic profile [52,53,54]. Our statistical analysis revealed a significant difference in the frequency of kidney dysfunction between patients with PAH and CTEPH, with a higher prevalence noted in the CTEPH group (35.71% vs. 2.86–8.57% in the PAH group). Although an improvement in the glomerular filtration rate was noted in the CTEPH group, this did not reach statistical significance.

In a study of 4635 catheterized patients, 40% suffered from chronic kidney disease (CKD), with 68% of these exhibiting PH. CKD was independently linked to an increased PH risk (OR = 1.4, 95% CI = 1.18–1.65). Mortality increases in line with the CKD stage and presence of PH, highlighting that CKD is a critical prognostic factor in PH patients and suggesting a potential role of CKD in developing pulmonary vascular disease [51]. Our findings regarding kidney dysfunction are consistent with those reported by Nickel et al., affirming the reliability of our results [17]. However, our study’s data on renal dysfunction did not align with data from the REVEAL registry, where a lower incidence of renal dysfunction was reported (4% of subjects with PH) [30].

Diuretic therapy, a fundamental aspect of PH management, was extensively utilized, with more than 87% of patients receiving diuretics throughout the study duration. This underscores the importance of monitoring renal function in PH patients, given the potential impact of diuretics on serum creatinine levels and eGFR.

Diuretics continue to be the primary treatment for managing congestion in CHF. The required intensity of diuretic therapy can differ based on the underlying causes and the severity of right heart failure, as well as other factors such as concurrent renal disease [55,56]. The dosage, combination, and duration of diuretic therapy may influence serum creatinine levels and eGFR. However, most studies do not provide comprehensive information regarding the specifics of diuretic usage. Among those studies that do report on diuretic use, there is substantial variability in the percentage of patients receiving diuretics, ranging from 50% to 90% [52,53].

Loop diuretics are indicated either alone or in combination with other medications, related to the patient’s congestive status. Careful administration is crucial to avoid excessive fluid loss, which could exacerbate reductions in cardiac output, cause symptomatic hypotension, or lead to renal impairment [57]. Mineralocorticoid receptor antagonists (i.e., spironolactone) can assist in preserving the potassium balance despite potassium losses; however, a study comparing high-dose versus low-dose spironolactone in acute heart failure did not show better clinical outcomes [58]. In our study, 82% to 94% of patients with PAH received loop diuretics and aldosterone antagonists, whereas spironolactone was recommended in 100% of CTEPH patients. Additionally, no statistically significant difference was observed between the groups in terms of diuretic therapy.

Regarding liver impairment, a progressive improvement was observed throughout the study period in PAH patients. Specifically, significant improvement was noted in patients with cholestasis syndrome, suggesting a favorable response to treatment over time.

The liver’s vulnerability to circulatory injury stems from its dual vascular supply and elevated metabolic demands. Acute heart failure with reduced cardiac output and shock can lead to ischemic hepatitis, a condition characterized by hepatocellular injury patterns marked by a rapid elevation in serum aminotransferases (aspartate and alanine transaminase, AST/ALT), as well as lactate dehydrogenase (LDH), frequently exceeding ten times the upper limit of normal [24,25]. Liver dysfunction among patients with chronic left heart disease (LHD) exhibits considerable variability but is predominantly associated with right heart hemodynamics—particularly central venous pressure—and RAP, rather than with left heart failure [27,28,29].

Elevated total bilirubin levels have been documented in 15 to 20% of individuals with PH. A study encompassing 404 patients with idiopathic PAH unveiled that direct bilirubin elevation was present in 37% of cases and was identified as an independent predictor of mortality. Multiple smaller-scale investigations involving well-defined cohorts of PAH patients reported mildly increased levels of total bilirubin, ALP, and GGT. Conversely, elevation in AST and ALT, collectively referred to as transaminases, appears to be less common, occurring in approximately 2% of cases [30,31,32].

In the REVEAL registry, it is noteworthy that 15% of patients exhibited elevated total bilirubin levels, contrasting with a prevalence of 4% for kidney dysfunction. Interestingly, while elevated bilirubin levels only showed a significant association with survival in the univariate analysis, kidney dysfunction retained its importance as a predictor of mortality in the multivariate analysis [30].

Elevations in bilirubin, GGT, AST, ALT, and ALP levels were prevalent at baseline and decreased slightly over the follow-up interval. However, these findings remained consistent with those reported by Ess et al., van Deursen et al. and Poelzl et al., who reported similar ranges of elevated liver enzymes in PH patients [27,28,29]. Particularly, only the values of AST and ALT obtained at inclusion approximate those published by Benza et al. (2%) [30].

Notably, while the total bilirubin levels showed no significant difference between the PAH and CTEPH groups at baseline and six months, a statistically significant decrease was observed at twelve months, highlighting potential differences in disease progression and treatment response. Only the data obtained at 12 months from the time of inclusion approximate those published in the REVEAL registry (15%) and by Takeda et al. (15 to 20%) [30,32]. Similarly, the AST and ALT levels did not differ significantly between the PAH and CTEPH groups throughout the study duration, indicating comparable hepatic involvement in both conditions.

According to a recent meta-analysis, bosentan was associated with the highest risk of hepatotoxicity [37]. This statement was not confirmed in the current study; however, a small proportion of patients undergoing bosentan therapy experienced elevated aminotransferase levels. Our findings are similar to those published by Segal et al. (7.6%) [38]. Additionally, a higher prevalence of elevated ALP was observed in patients with CTEPH compared with PAH.

Mild elevations in ALP are found in 17% to 77% of patients with stable CHF. In our study, elevations of ALP were detected in 18.36% of patients at the time of inclusion, 24.49% at six months, and 20.41% at twelve months. These values are consistent with those reported in the literature [27,28,29]. At the time of inclusion in this study, the ALP levels were not significantly different between the PAH and CTEPH groups. However, at six and twelve months, the ALP levels in CTEPH cases were significantly higher than those with PAH, but no correlation was found between ALP levels and the treatment of patients with CTEPH.

A recent paper disclosed that reduced blood lipid levels in PH, specifically total cholesterol (TC) and triglycerides (TG), are linked with RV dilation, RV dysfunction, abnormal RV metabolism, and higher levels of NT-proBNP. Given that RV failure plays a crucial role in determining the severity and prognosis of PH, this association indicates that targeting the lipid metabolic pathway could be a significant therapeutic approach to enhance RV function and improve the clinical outcomes in PH [59]. This association has been well documented in conditions such as heart failure and among aging individuals [60]. Restoring TC levels to within normal ranges is hypothesized to mitigate systemic inflammation and address malnutrition-related impacts, potentially leading to enhanced patient outcomes in these contexts.

In left heart disorders such as coronary artery disease, hypertension, and atherosclerosis, elevated circulating cholesterol is recognized as a major cardiovascular risk factor. Conversely, in PH, these lipid levels are decreased, which aligns with our findings [41,61]. Previous studies have consistently reported that TG levels remain largely unchanged in PH, a finding that is supported by our study. However, certain research results suggest an elevation in TG levels among patients with idiopathic pulmonary arterial hypertension, possibly influenced by specific patient characteristics [40,60,62].

In our study, there was no statistically significant difference observed in the levels of TC and serum TG between the studied groups of PH patients. Additionally, the specific PH drug regimen did not show any recognizable alteration in lipid metabolism.

Considering the specific pharmacologic regimens across the three specified time points revealed notable trends. Specifically, at inclusion in this study, patients were treated with a combination of drugs depending on their PH group, including PAH-specific therapies and anticoagulants in the CTEPH group.

Sildenafil, the first PDE5i studied in PH, demonstrates a range of nephroprotective effects. These encompass a reduction in systemic blood pressure, the amelioration of tissue injury following ischemia–reperfusion events, the amelioration of kidney damage induced by contrast and cyclosporine, and the attenuation of glomerulosclerosis aggravated by the presence of type 2 diabetes mellitus (T2DM) [13].

Post-marketing data revealed that 7.6% of patients on bosentan experienced elevated aminotransferase levels. The liver injury induced by bosentan appears to be dose-dependent. While hepatocellular injury expressed by elevated AST/ALT is the most common side effect during bosentan therapy, instances of cholestatic liver injury with increased ALP levels have also been reported [38].

Throughout the study period, there was a trend of medication regimens being adapted, frequently reflecting changes made in response to clinical outcomes, disease progression, and adverse/side effects. In the PAH group, there was a steady introduction of upfront PAH-specific therapies. Conversely, in the CTEPH group, while PAH-specific therapies might be initiated in cases of concurrent PAH or inoperable CTEPH, anticoagulation remained a cornerstone therapy throughout the study duration. These shifts in medication patterns underscore the dynamic nature of PH management and the importance of individualized treatment strategies that are tailored to a patient’s response and disease trajectory.

The present study is characterized by inherent limitations that warrant consideration in the interpretation of our findings. Firstly, its single-center design and the modest sample size (*n* = 49 cases) inherently constrain the generalizability of our results to broader populations. Consequently, the given data necessitate careful and personalized examination during their analysis and interpretation, as well as validation through multicenter studies with larger cohorts to enhance the robustness of our conclusions. Furthermore, potential confounding factors, including dietary patterns, lifestyle variables, other medications, and comorbidities, add complexities that could influence the observed parameters. Other vasodilators such as prostaglandin (epoprostenol) and the synthetic stable tricyclic analog of prostacyclin (treprostinil) were not available at the time of this study. Additionally, the absence of access to pulmonary endarterectomy (PEA) and balloon pulmonary angioplasty (BPA)—therapies that were unavailable in our country at the time of this study—along with the limited number of cases undergoing these interventions nationwide, constitutes a significant limitation of this study. Discreet differences in group results could be due to the small number of patients or the nature of this study.

## 5. Conclusions

The present study offers insights into kidney dysfunction, hepatic impairment, and lipid metabolism abnormalities in a limited cohort of 49 patients with PAH (*n* = 35) and CTEPH (*n* = 14 cases), over a 12-month observational period. The findings highlight the need for tailored management approaches for different PH subtypes.

Kidney dysfunction was consistently more prevalent in the CTEPH group than the PAH group, with a notable albeit not statistically significant improvement in the glomerular filtration rate observed among CTEPH patients. Furthermore, the prevalence of kidney dysfunction was statistically significantly higher among CTEPH patients compared to PAH patients at all three study time points.

Hepatic impairment showed a statistically significant improvement in the PAH cohort, especially in relation to cholestasis syndrome, while no significant changes were observed in the CTEPH group. This study also identified elevated levels of ALP and GGT in CTEPH patients at all time points, with ALP levels showing significant differences between the two groups. Notably, the initiation of targeted therapy for pulmonary hypertension resulted in a statistically significant improvement in hepatic impairment within the PAH group.

Lipid metabolism disorders were less frequent, with no significant differences between the groups, likely influenced by the use of lipid-lowering agents.

Furthermore, improvements in 6MWT performance were more pronounced in CTEPH patients, while NT-proBNP levels remained significantly higher in this group compared to PAH patients. Also, the four-strata risk assessment did not reveal statistically significant differences in risk scores between PAH and CTEPH groups across the study period.

In conclusion, our study offers comprehensive insights into the kidney, liver, and lipid metabolism profiles in PAH and CTEPH patients, emphasizing the intricate interplay of comorbidities and treatment responses. These findings underscore the necessity for personalized management strategies and tailored interventions. Multicenter studies are needed to validate findings to enhance patient outcomes in this specific population.

## Figures and Tables

**Table 1:** Clinical characteristics of the study cohort at baseline and during follow-up (6 and 12 months).

General Characteristics	PAH Patients	CTEPH Patients	*p*-Value
	(*n* = 35)	(*n* = 14)	
Sex (female)	22 (62.85)	8 (57.1)	ns
Age (years)	47 (27–63)	63.5 (53.75–69)	0.0142
BMI (kg/m^2^)	24.68 (21.09–29.68)	31.07 (26.31–32.52)	0.0149
Heart rate (bpm)	85 (70–92)	76 (69.75–93.75)	ns
Systolic BP (mmHg)	120 (110–130)	117.5 (110–131.3)	ns
Diastolic BP (mmHg)	70 (60–85)	80 (70–82.5)	ns
Mean BP (mmHg, IQR)	86.67 (78.33–100)	91.67 (83.33–97.92)	ns
Time from the onset of symptoms to diagnosis (months, median, IQR)	12.00 (6.00–24.00)	12.00 (5.25–17.25)	ns
6-MWD (m)			
At inclusion	400 (255–455)	300 (95–387)	0.0345
At 6 months	430 (283–465)	367 (195–422)	ns
At 12 months	439 (293–473.5)	386.5 (253.8–492.5)	ns
NT-proBNP (pg/mL, median, IQR)			
Baseline	400 (145–2619)	1243 (340.5–5843)	ns
At 6 months	1106 (263.5–2128)	1666 (528–3997)	ns
At 12 months	485.5 (144.3–1402)	1465 (388–4626)	0.045
Right heart catheterization data			
mPAP (mmHg)	51.22 ± 17.14	42.50 ± 10.67	ns
PAWP (mmHg)	11.00 (6.50–13.00)	11.50 (8.75–14.50)	ns
PVR (WU)	10.00 (4.01–14.00)	9.50 (5.50–12.00)	ns
CO (l/min)	5.14 ± 2.07	4.57 ± 2.04	ns
CI (L/min/m^2^)	2.95 (2.19–3.70)	2.38 (1.90–2.65)	ns
Proximal vessel disease (*n*, %)	N/A	7 (50)	

6-MWD, 6 min walking distance; BMI, body mass index; BP, blood pressure; bpm, beats per minute; CI, cardiac index; CO, cardiac output; CTEPH, chronic thromboembolic pulmonary hypertension; IQR, interquartile range; mPAP, mean pulmonary arterial pressure; *n*, number of patients; NT-proBNP, N-terminal pro B-type natriuretic peptide; ns, statistically insignificant; PAH, pulmonary arterial hypertension; PAWP, pulmonary artery wedge pressure; PVR, pulmonary vascular resistance; WU, Wood units. Cardiac output was calculated using Fick principle.

**Table 2:** Main PH-related factors of kidney, hepatic, and lipid metabolism parameters in our studied patients.

	PAH Patients		CTEPH Patients		*p*-Value
Median WHO functional class (IQR)					
Baseline	3.00 (2.00–3.00)		2.50 (2.00–3.00)		ns
At 12 months	2.00 (2.00–3.00)		2.00 (2.00–3.00)		ns
WHO functional class	Baseline	At 12 months	Baseline	At 12 months	
I (*n*, %)	2 (5.72)	2 (5.72)	1 (7.15)	2 (14.28)	
II (*n*, %)	14 (40)	17 (48.57)	6 (42.85)	6 (42.85)	
III (*n*, %)	18 (51.43)	16 (45.71)	5 (35.72)	5 (35.72)	
IV (*n*, %)	1 (2.85)	0	2 (14.28)	1 (7.15)	
Median severity of right heart failure (IQR)					
Baseline	2.00 (1.00–2.00)		2.00 (1.00–2.00)		ns
At 12 months	2.00 (1.00–2.00)		2.00 (1.25–2.00)		ns
Severity of right heart failure	Baseline	At 12 months	Baseline	At 12 months	
Normal (*n*, %)	19 (54.28)	25 (71.42)	6 (42.85)	9 (64.28)	
Mild/Moderate (*n*, %)	13 (37.14)	10 (28.58)	5 (35.72)	4 (28.57)	
Severe (*n*, %)	3 (8.58)	0	3 (21.43)	1 (7.15)	
Duration of the PH (months, IQR)	30.00 (13.00–113.50)		20.00 (14.50–34.00)		ns

CTEPH, chronic thromboembolic pulmonary hypertension; IQR, interquartile range; *n*, number of patients; ns, statistically insignificant; PAH, pulmonary arterial hypertension; PH, pulmonary hypertension; WHO, World Health Organization.

**Table 3:** Main non-PH-related factors of kidney, hepatic, and lipid metabolism parameters in our studied patients.

	PAH Patients	CTEPH Patients	*p*
T2DM (*n*, %)	7 (20.00)	5 (35.71)	ns
HTN (*n*, %)	11 (31.42)	7 (50.00)	ns
CHF (*n*, %)	20 (57.1)	10 (71.42)	ns
Coronary heart disease (*n*, %)	1 (2.85)	2 (14.28)	ns
Deep vein thrombosis (*n*, %)	0	14 (100.0)	<0.0001
Atrial fibrillation (*n*, %)	11 (31.42)	6 (42.85)	ns
Thyroid disease (*n*, %)	11 (31.42)	3 (21.42)	ns
Obstructive sleep apnea (*n*, %)	2 (5.71)	3 (21.42)	ns
Lung disease—other than COPD and asthma (*n*, %)	12 (34.28)	4 (28.57)	ns
COPD (*n*, %)	4 (11.42)	1 (7.14)	ns
Asthma (*n*, %)	4 (11.42)	2 (14.28)	ns
SARS-CoV-2 (*n*, %)	12 (34.28)	4 (28.57)	ns

CHF, congestive heart failure; COPD, chronic obstructive pulmonary disease; CTEPH, chronic thromboembolic pulmonary hypertension; HTN, hypertension; *n*, number of patients; ns, statistically insignificant; PAH, pulmonary arterial hypertension; PH, pulmonary hypertension; T2DM, type 2 diabetes mellitus.

**Table 4:** Characteristics of all patients, comparing the three time points of this study (baseline, 6 months, and 12 months).

Parameters	PAH Patients (*n* = 35)	CTEPH Patients (*n* = 14)	*p*
Kidney dysfunction (*n*, %)			
Baseline	1 (2.86)	5 (35.71)	0.0052
At 6 months	3 (8.57)	5 (35.71)	0.0333
At 12 months	1 (2.86)	3 (21.43)	0.0052
Creatinine (mg/dL)			
Baseline	0.83 (0.75–0.96)	1.08 (0.96–1.35)	0.0011
At 6 months	0.83 (0.72–0.96)	0.94 (0.82–1.37)	0.031
At 12 months	0.84 (0.73–0.96)	0.91 (0.86–1.23)	ns
eGFR (mL/min/1.73 m^2^)			
Baseline	94.66 ± 22.88	67.84 ± 19.7	0.0004
At 6 months	96.6 ± 22.91	69.57 ± 26.37	0.0008
At 12 months	96.98 ± 21.12	74.48 ± 24.66	0.0024
Hepatic impairment (*n*, %)			
Baseline	17 (48.57)	11(78.57)	ns
At 6 months	16 (45.71)	10(71.43)	ns
At 12 months	10 (28.57)	11(78.57)	0.0031
Total bilirubin (mg/dL)			
Baseline	0.9 (0.6–1.4)	1.2 (0.77–1.72)	ns
At 6 months	0.8 (0.5–1.3)	0.9 (0.67–1.55)	ns
At 12 months	0.7 (0.5–1.1)	0.9 (0.77–1.52)	0.0384
GGT (U/L)			
Baseline	30 (22–57)	56 (35.5–79.5)	0.013
At 6 months	29 (23–66)	59.5 (29.75–86)	0.0424
At 12 months	26 (21–43)	59.5 (31.75–180)	0.0031
AST (U/L)			
Baseline	22 (17–28)	21 (16.75–27)	ns
At 6 months	21 (15–25)	18 (17.5–25.5)	ns
At 12 months	20 (16–27)	22 (19–25.25)	ns
ALT (U/L)			
Baseline	15 (11–23)	18 (13.75–27.5)	ns
At 6 months	16 (12–23)	17.5 (12.5–26.75)	ns
At 12 months	16 (10–23)	19 (16.5–26.75)	ns
ALP (U/L)			
Baseline	196 (173–236)	247.5 (151.8–351.3)	ns
At 6 months	186 (149–288)	266 (195.8–400.5)	0.05
At 12 months	187 (156–257)	240.5 (202.3–375)	0.0169
Lipid metabolism abnormalities			
Baseline	6 (17.14)	5 (35.71)	ns
At 6 months	3 (8.57)	3 (21.43)	ns
At 12 months	8 (22.86)	4 (28.57)	ns
Total cholesterol (mmol/L)			
Baseline	4.29 ± 0.95	4.59 ± 1.75	ns
At 6 months	4.08 ± 0.65	4.31 ± 1.44	ns
At 12 months	4.15 ± 0.94	4.23 ± 1.16	ns
Triglycerides (mmol/L)			
Baseline	1.07 (0.78–1.35)	1.15 (0.91–1.84)	ns
At 6 months	0.97 (0.71–1.16)	1.23 (0.87–1.62)	ns
At 12 months	1.01 (0.63–1.43)	1.39 (0.91–1.89)	ns

ALP, alkaline phosphatase; ALT, alanine transaminase; AST, aspartate transaminase; eGFR, estimated glomerular filtration rate; GGT, gamma-glutamyl transferase; *n*, number of patients; ns, statistically insignificant.

**Table 5:** Drug regimens in the studied group.

Medication	PAH (*n* = 35)	CTEPH (*n* = 14)	*p*
ERA			
Baseline	5 (14.29)	2 (14.29)	ns
At 6 months	3 (8.57)	3 (21.43)	ns
At 12 months	2 (5.71)	2 (14.29)	ns
PDE5i			
Baseline	14 (40)	5 (35.71)	ns
At 6 months	0 (0)	6 (17.14)	
At 12 months	0 (0)	6 (17.14)	
ERA + PDE5i			
Baseline	15 (42.86)	5 (35.71)	ns
At 6 months	25 (71.43)	8 (57.14)	ns
At 12 months	26 (74.29)	8 (57.14)	ns
SGCSs			
Baseline	1 (2.86)	3 (21.43)	ns
At 6 months	1 (2.86)	4 (28.57)	0.0194
At 12 months	1 (2.86)	5 (35.71)	0.0052
ACEI/ARB			
Baseline	5 (14.71)	0 (0)	
At 6 months	2 (5.71)	0 (0)	
At 12 months	3 (8.57)	0 (0)	
Loop diuretics			
Baseline	29 (82.86)	14 (100)	ns
At 6 months	32 (91.42)	14 (100	ns
At 12 months	32 (91.42)	14 (100	ns
Spironolactone			
Baseline	29 (82.86)	14 (100)	ns
At 6 months	33 (94.29)	14 (100	ns
At 12 months	33 (94.29)	14 (100	ns
Statins			
Baseline	7 (20)	4 (28.57)	ns
At 6 months	5 (14.29)	5 (35.71)	ns
At 12 months	5 (14.29)	6 (42.86)	ns
Beta-blockers			
Baseline	11 (31.43)	10 (71.43)	0.0233
At 6 months	14 (40)	8 (57.14)	ns
At 12 months	16 (45.71)	8 (57.14)	ns
I_f_ channel blocker			
Baseline	9 (25.71)	3 (21.43)	ns
At 6 months	8 (22.86)	3 (21.43)	ns
At 12 months	7 (20)	2 (14.29)	ns

ACEI/ARB, angiotensin-converting enzyme inhibitor/angiotensin receptor blocker; ERA, endothelin receptor antagonist; PDE5i, phosphodiesterase type 5 inhibitor; SGCSs, soluble guanylate cyclase stimulators.

## Data Availability

The data that support the findings of this study are available on request from the corresponding author. The data are not publicly available due to general data protection regulations.
